# Teaching Endotracheal Intubation Using a Cadaver Versus a Manikin-based Model: a Randomized Controlled Trial

**DOI:** 10.5811/westjem.2019.10.44522

**Published:** 2019-12-09

**Authors:** Ryan Pedigo, Juliana Tolles, Daena Watcha, Amy H. Kaji, Roger J. Lewis, Elena Stark, Jaime Jordan

**Affiliations:** * Harbor-UCLA Medical Center, Department of Emergency Medicine, Torrance, California; † Los Angeles Biomedical Research Institute, Torrance, California; ‡ David Geffen School of Medicine at UCLA, Department of Emergency Medicine, Los Angeles, California; § David Geffen School of Medicine at UCLA, Department of Pathology and Laboratory Medicine, Los Angeles, California; ¶ UCLA Ronald Reagan Medical Center, Department of Emergency Medicine, Los Angeles, California

## Abstract

**Introduction:**

The optimal method to train novice learners to perform endotracheal intubation (ETI) is unknown. The study objective was to compare two models: unembalmed cadaver vs simulation manikin.

**Methods:**

Fourth-year medical students, stratified by baseline ETI experience, were randomized 1:1 to train on a cadaver or simulation manikin. Students were tested and video recorded on a separate cadaver; two reviewers, blinded to the intervention, assessed the videos. Primary outcome was time to successful ETI, analyzed with a Cox proportional hazards model. Authors also compared percentage of glottic opening (POGO), number of ETI attempts, learner confidence, and satisfaction.

**Results:**

Of 97 students randomized, 78 were included in the final analysis. Median time to ETI did not differ significantly (hazard ratio [HR] 1.1; 95% CI [confidence interval], 0.7–1.8): cadaver group = 34.5 seconds (interquartile ratio [IQR]: 23.3–55.8) vs manikin group = 35.5 seconds (IQR: 23.8–80.5), with no difference in first-pass success (odds ratio [OR] = 1; 95% CI, 0.1–7.5) or median POGO: 80% cadaver vs 90% manikin (95% CI, −14–34%). Satisfaction was higher for cadavers (median difference = 0.5; p = 0.002; 95% CI, 0–1) as was change in student confidence (median difference = 0.5; p = 0.03; 95% CI, 0–1). Students rating their confidence a 5 (“extremely confident”) demonstrated decreased time to ETI (HR = 4.2; 95% CI, 1.0–17.2).

**Conclusion:**

Manikin and cadaver training models for ETI produced similar time to ETI, POGO, and first-pass success. Cadaver training was associated with increased student satisfaction and confidence; subjects with the highest confidence level demonstrated decreased time to ETI.

## INTRODUCTION

Endotracheal intubation (ETI) is a vital skill for many medical practitioners, including those in emergency medicine (EM), critical care, and anesthesia, but there is a significant learning curve in gaining proficiency.^1^ The rate of successful ETI for inexperienced personnel on their first attempt using direct laryngoscopy may be as low as 50%.^2^ A systematic review found that to achieve at least a 90% success rate within two attempts under optimal elective conditions, a minimum experience of 50 ETIs was required.^3^ Teaching ETI to novices in settings such as the emergency department or intensive care unit is potentially unsafe for critically ill patients, as much higher complication rates in these populations have been reported.^4^ Teaching airway management to inexperienced students in a more controlled setting, such as the operating room, is not always practical because of patient safety concerns and the presence of multiple learners with relatively limited numbers of patients.^5^

Historically, many students have learned ETI on simulation manikins. The reported advantages of simulation training include the fact that it allows for simultaneous teaching of ETI to many individuals and less pressure on the student, without danger to patients.^6^ However, it is unclear whether learning ETI on manikins sufficiently prepares novices for intubating patients. It is impossible for the rigid construction of the plastic manikin airway to reproduce human anatomy with high fidelity.^7^ Previous studies found that the use of a fresh frozen cadaver or lightly embalmed cadaver for training ETI achieves greater realism and that learners prefer cadavers to a simulation manikin, but these studies did not assess for objective outcome data.^8^,^9^ A previous study showed that a cadaver-based airway lab can improve the ETI success rate of critical care medicine fellows, but this study did not have a control group.^10^

The optimal model for providing ETI training to novice learners is currently unknown. The objective of this study was to compare two training models, unembalmed cadaver vs simulation manikin, on ETI procedural competency in fourth-year medical students as measured by time to successful ETI. We also sought to compare percentage of glottic opening (POGO) viewed, number of attempts needed to achieve successful ETI, as well as learner confidence and satisfaction. We hypothesized that training using an unembalmed cadaver model would be the more effective model.

## METHODS

### Study setting and participants

This study took place in the Gross Anatomy Laboratory in the Center for Health Sciences at the David Geffen School of Medicine at the University of California in Los Angeles between July 2015–March 2017. Fourth-year medical students enrolled in the EM sub-internship or emergency procedures elective during the study period were eligible to participate. This included students from the David Geffen School of Medicine at UCLA, as well as outside rotating students from over 20 institutions. Students with a pre-existing physical limitation that would preclude them from performing ETI were excluded.

This study was approved by the institutional review board (IRB) of the Los Angeles Biomedical Research Institute at Harbor-UCLA Medical Center. Students were consented with a standard IRB consent and were permitted to withdraw their participation at any time.

### Study design

This was a prospective, randomized controlled trial. Prior to the intervention, we collected baseline characteristics of the study subjects, including (1) self-reported number of prior ETI attempts, (2) number of successful ETI, and (3) perceived confidence in performing ETI using a 5-point Likert-type scale (1: not at all confident, 2: slightly confident, 3: moderately confident, 4: very confident, 5: extremely confident). On the day of the intervention, all students received an airway lecture from the course director (author RP) covering the general approach to airway management and appropriate patient positioning. We used stratified, permuted block randomization to randomize students to manikin or unembalmed cadaver training to ensure equal baseline ETI experience between groups.

**Educational Research Capsule Summary**

What do we already know about this issue?

*Endotracheal intubation has a substantial learning curve and various teaching modalities have been employed to teach this critical skill*.

What was the research question?

Is an unembalmed cadaver or a simulation manikin a more effective model for teaching endotracheal intubation?

What was the major finding of the study?

*Both unembalmed cadaver and simulation manikin were similarly effective, but learners prefer the cadaver*.

How does this improve population health?

*Understanding the optimal modality of teaching endotracheal intubation can improve patient outcomes for this critical procedure*.

Three categories based on number of prior ETI attempts (< 10: low experience, 10–24: medium experience, 25+: high experience) were selected based on existing learning curve data.^1^ Students then received a 30-minute hands-on training session with their assigned model (unembalmed cadaver or manikin) delivered by an EM faculty member practicing direct laryngoscopy. We used new Laerdal airway management trainers as our simulation manikins, as previous studies suggested that this was rated as the most realistic and highest-performance manikin.^8^,^11^

To mitigate any potential differences between instructor teaching effectiveness, the instructors were also randomly assigned to the student groups via a coin toss. The instructor-led workshops were pragmatic and reflected the variation in teaching methods that would be present in other institutions; we did not require a specific modality of bedside hands-on ETI experience. After the training session, we again surveyed students on their perceived confidence in ETI using the same 5-point Likert-type scale. Satisfaction with the training method was assessed with a 5-point Likert-type scale (1: very dissatisfied, 2: dissatisfied, 3: unsure, 4: satisfied, 5: very satisfied).

To evaluate procedural competency, we assessed student performance of ETI on an unembalmed cadaver on which neither group had previously used in training. We used an unembalmed cadaver as the testing medium for all learners to eliminate or mitigate any between-group differences in airway difficulties that could confound our results. In addition, an unembalmed cadaver has previously been shown to have a high degree of airway realism compared to patients.^8^ Students performed ETI using direct laryngoscopy with a Karl Storz C-Mac with available Mac 3 and Mac 4 size attachments. Subjects did not have access to the monitor during the ETI attempt; the video screen was turned away from the subject but was recorded to obtain the necessary data. After the student verbalized that they felt their ETI attempt was successful, RP verified correct placement of the endotracheal tube (ETT). If the ETT was not placed successfully, a failed ETI attempt was noted and the student had the opportunity to attempt again. RP recorded the total number of ETI attempts.

Two reviewers (JJ and JT), who were blinded to the intervention, reviewed the video recordings with student faces and any identifying information blurred and rated the time to successful ETI and POGO achieved for all participants.

### Outcome measures

The primary outcome was time to successful ETI, defined as time from picking up the laryngoscope to the time in which the student verbalized successful ETI on an unembalmed cadaver.

Secondary outcomes included the following:

1. POGO achieved
2. Number of ETI attempts
3. Changes in perceived confidence after practice session
4. Whether or not increased confidence translated into improved ETI performance
5. Satisfaction with the teaching modality.

### Data analysis

The primary outcome of time to ETI success was analyzed using a Cox proportional hazards model, in which we included prior ETI experience and date of rotation as covariates in the analysis. These covariates were chosen because increased experience has been shown to decrease time to ETI (although we did mitigate this with our randomization scheme), and there may have been differences in difficulty of cadaver airway anatomy in each different session. The mean POGO and time to ETI recorded by the two reviewers’ scores was calculated. We analyzed the difference in mean reviewer POGO score between manikin and cadaver groups using a bootstrap resampling method with 10,000 iterations. Correlation coefficients and a Bland-Altman plot were used to evaluate for correlation and presence of bias in the video analysis for the POGO score and time to ETI. We compared changes in confidence score and overall satisfaction using the Wilcoxon rank-sum test with the confidence interval (CI) calculated with the Hodges-Lehmann estimator. For this analysis, CIs were rounded to the nearest integer to reflect the precision that could be expected from our sample size. We analyzed the association between student confidence and time to ETI using a Cox proportional hazards model. All analyses were conducted using the R software for statistical computing, version 3.3.1, employing the Survival package and Boot package.

## RESULTS

**Figure 1:**
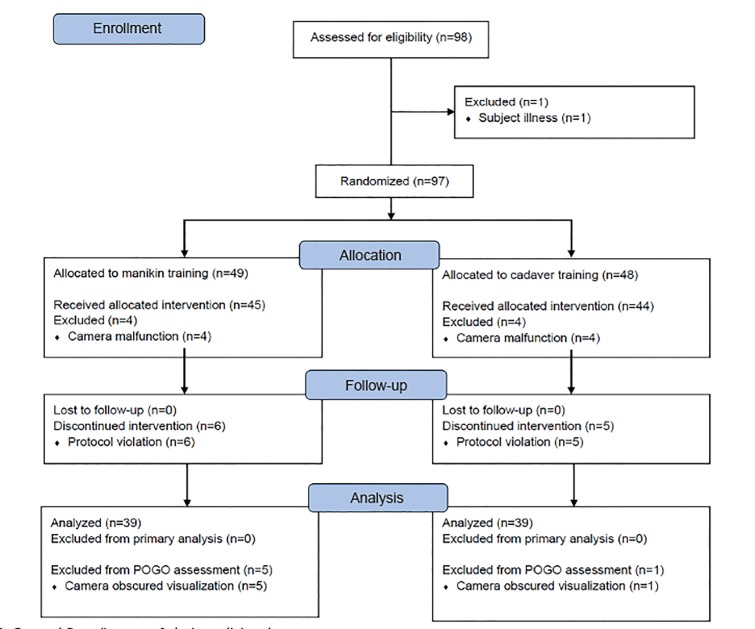
Consort flow diagram of study participants.

We assessed 98 medical students for eligibility (Figure 1). We excluded one student due to illness. We randomized 97 students to cadaver or manikin training. The stratified randomization scheme was successful in distributing ETI experience similarly between the two groups. We could not obtain data for one session due to a camera malfunction; this excluded one group of eight students from the analysis. A protocol violation affected another group of 11 subjects, where the cadaver arm subjects inadvertently also practiced intubating the cadaver intended for testing; thus, we excluded data from these subjects. Data from 78 subjects were included in the final analysis. We could not judge the POGO for six of these cases, due to the camera becoming obscured.

**Figure 2:**
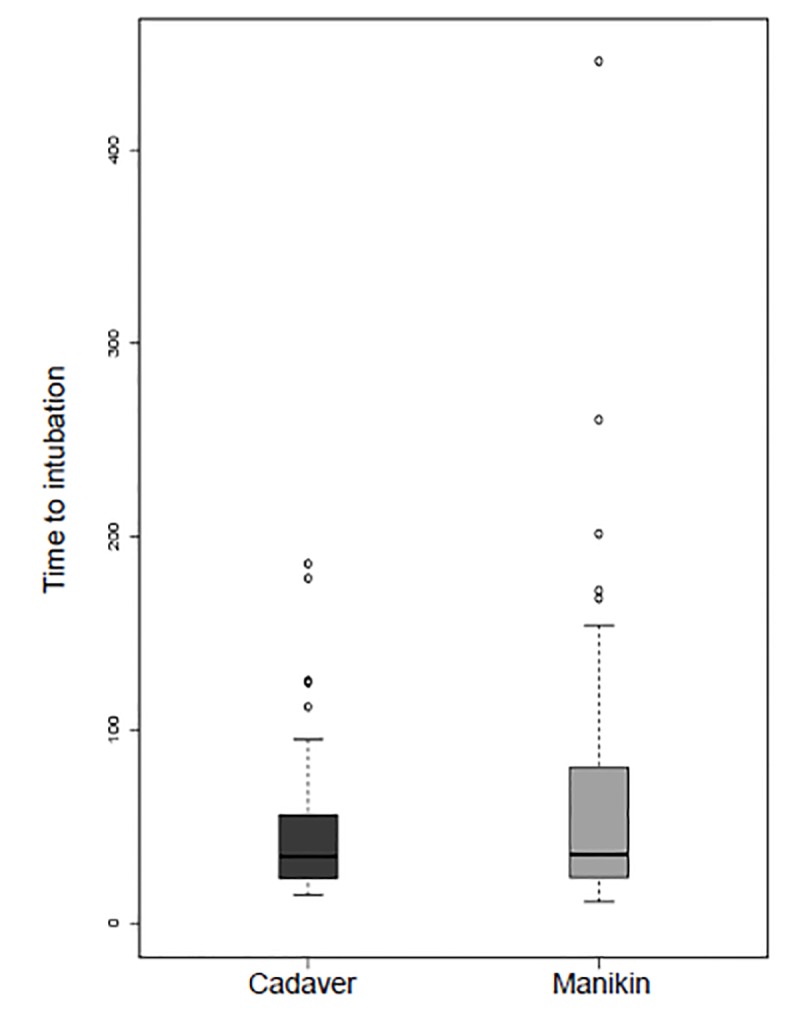
Time to successful endotracheal intubation after subjects were randomized to a manikin or cadaver training model.

The median time to ETI for the cadaver group was 34.5 seconds (interquartile range [IQR]: 23.3–55.8 seconds) and the median time to ETI for the manikin group was 35.5 seconds (IQR: 23.8–80.5 seconds) (Figure 2).

The time to ETI did not differ between the two groups (hazard ratio [HR] 1.1 for ETI in the cadaver group; 95% CI, −0.7–1.8). The HR in this case describes ratio of the rate of intubation per unit time between the two groups. The correlation coefficient between the two reviewers’ assessments of time to ETI was 0.99. There was no difference in first-pass success between groups (odds ratio [OR]=1; 95% CI, 0.1–7.5). We performed an exploratory analysis for an interaction between learner experience and treatment group to see whether any experience-level subgroup had benefit from either a cadaver or manikin training model, but no significant effect was found.

**Figure 3:**
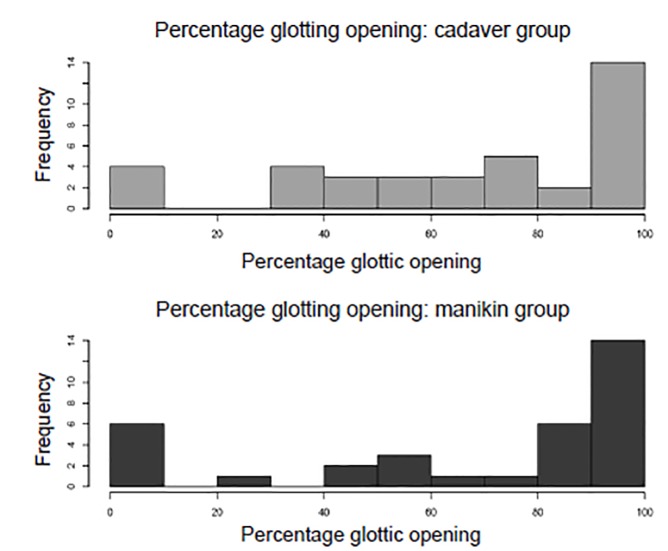
Percentage of glottic opening achieved after subjects were randomized to a manikin or cadaver training model.

The median difference in POGO was not significantly different between treatment groups. The cadaver group had a median glottic opening of 80% and the manikin group 90% (10% median difference; 95% CI, −14–34%). We compared the median values because the observed distribution of scores was bimodal; visual inspection of the distribution (Figure 3) also reveals the distribution of scores to be very similar. The correlation coefficient between reviewer assessments of POGO was 0.98. A Bland-Altman plot of the difference between reviewer scores vs average reviewer score did not demonstrate systematic bias (Supplemental Figure 1).

**Figure 4:**
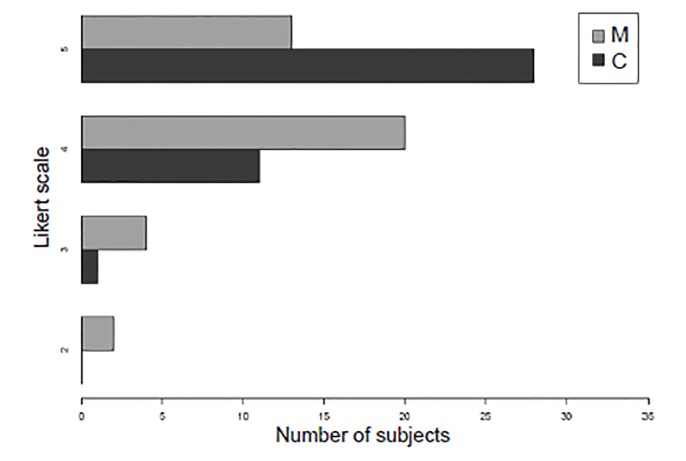
Subject satisfaction with each training model on a 5-point Likert-type scale. *M*, manikin model; *C*, cadaver model.

**Figure 5:**
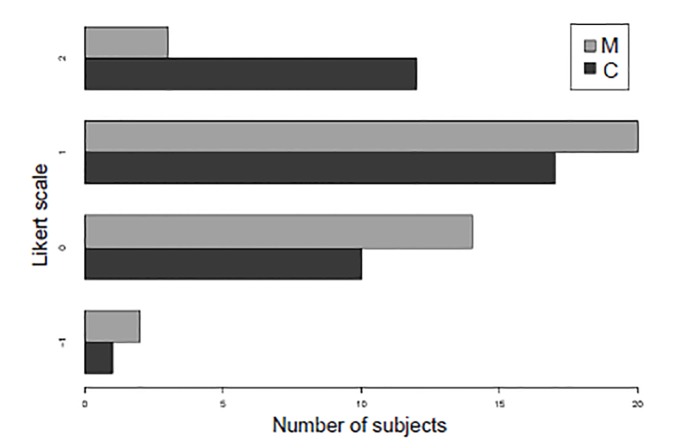
Subject confidence change before training model vs after training model on a 5-point Likert scale. *M*, manikin model; *C*, cadaver model.

The median subject satisfaction with the training exercise was higher for the cadaver-training group (median difference = 0.5 points; p = 0.002; 95% CI, 0–1) (Figure 4). Change in subjects’ confidence in ETI skills was also greater for the cadaver-training group than the manikin-training group (median difference = 0.5; p = 0.03; 95% CI, 0–1) (Figure 5). The students who rated their confidence after the teaching intervention as a 5 (“extremely confident”) also demonstrated a decreased time to successful ETI (HR = 4.2; 95% CI, 1.03–17.2).

## DISCUSSION

Our study is, to our knowledge, the largest randomized trial comparing unembalmed cadaver training to simulation manikin training for ETI. Both manikin- and cadaver-based training are highly effective in teaching ETI with no difference found in outcomes of time to successful ETI, number of attempts, or POGO score. There was a relationship with prior ETI experience and time to successful ETI, which is consistent with prior literature.^1^,^3^ We stratified by prior experience in our randomization scheme, so this did not affect our results.

Learner satisfaction was higher among those trained using a cadaver compared to a manikin. This may be due to the fact that when compared to the manikin, the cadaver provides a more realistic airway as well as normal tissue handling characteristics.^7^ Additionally, the use of a cadaver for learning purposes is novel, and this may also increase satisfaction. The subject-perceived increase in confidence in ETI was also greater in those trained on the cadaver model. This is not surprising, as studies have shown that cadaver-based procedural teaching increases learner confidence in performing other procedures such as central lines, pericardiocentesis, thoracentesis, and bag-valve-mask ventilation.^9^,^12^,^13^

Students who self-reported their confidence as a “5” after being exposed to the training method also demonstrated a decreased time to ETI. As only four students chose this level of confidence, we would recommend further studies before using confidence as a surrogate measure for performance in ETI.

## LIMITATIONS

Our study has several limitations. First, it is based upon results from a single center. However, the sub-internship enrollment comprised a large number of visiting students, and thus our results are likely generalizable to other institutions. Our subjects were all fourth-year medical students going into a variety of specialties. It is unclear whether our results are applicable to other types of ETI learners, but it is unlikely that medical students would be intrinsically much different from other novice learners for this skill. The subjects randomized to the cadaver training were exposed to an unembalmed cadaver, which may have theoretically made them more effective at intubating the other unembalmed test cadaver.

There was limited literature to guide expected ETI times and variability of times for novice learners; therefore, an a priori power calculation was not performed. A post hoc calculation demonstrated that our sample size produced 80% power to detect a 30-second difference in time to ETI from 40 seconds average time for an experienced provider, a time chosen based on data from a randomized trial of intubation of traumatic injury patients.^14^ We considered a 30-second difference in time to ETI to be the threshold of clinical significance based on time to desaturation in previous studies. For example, the ENDAO trial showed low rates of desaturation with a mean intubation time of 64 seconds when patients were preoxygenated and used apneic oxygenation in an emergent intubation setting.^15^ Our study showed median intubation times of 35 seconds. This is congruent with another study evaluating EM resident ETI times, which found a mean time to intubation of 32.7 seconds when residents were standing, which is the same position used by the medical students.^16^ Thus, although a smaller true difference may exist, it would be unlikely to be clinically significant, given that even the most extreme difference would be within previously-described safe apnea time limits.

Additionally, we excluded 11 subjects because of a protocol violation and eight students due to camera malfunction. However, since the students were eliminated from both groups (cadaver and manikin) on both dates, it is unlikely to have affected our results. Finally, obscuration of the camera prevented the assessment of POGO scores in seven students but did not affect the assessment of other outcomes.

## CONCLUSION

Unembalmed cadavers and simulation manikins had similar effectiveness in teaching ETI to fourth-year medical students based on time to ETI and POGO score. However, students training with unembalmed cadavers had higher degrees of satisfaction and greater increases in subjective confidence levels in performing the procedure. Students who expressed the highest confidence level also demonstrated faster ETI times.

## Supplementary Information
