# Supplementary material for: Teaching Endotracheal Intubation Using a Cadaver Versus a Manikin-based Model: a Randomized Controlled Trial
Source: West J Emerg Med. 2019 Dec 9;21(1):108–14. doi: 10.5811/westjem.2019.10.44522 (PMC6948684; doi:10.5811/westjem.2019.10.44522)
Supplement: Supplementary file 1 [file wjem-21-108-s001.docx]

Supplemental Figure 1. The Bland-Altman blot demonstrates minimal bias and good agreement between reviewer scores of percentage of glottic opening (POGO). The line at x = -0.008 marks the mean of differences between reviewer times and represents a bias of -0.8% for reviewer JJ as compared to reviewer JT.
